# Supplementary material for: Evidence supported by Mendelian randomization: impact on inflammatory factors in knee osteoarthritis
Source: Front Med (Lausanne). 2024 May 28;11:1382836. doi: 10.3389/fmed.2024.1382836 (PMC11165061; doi:10.3389/fmed.2024.1382836)
Supplement: Supplementary file 1 [file Data_Sheet_1.ZIP › data/GCST90274780_5e6/780.forest.pdf]

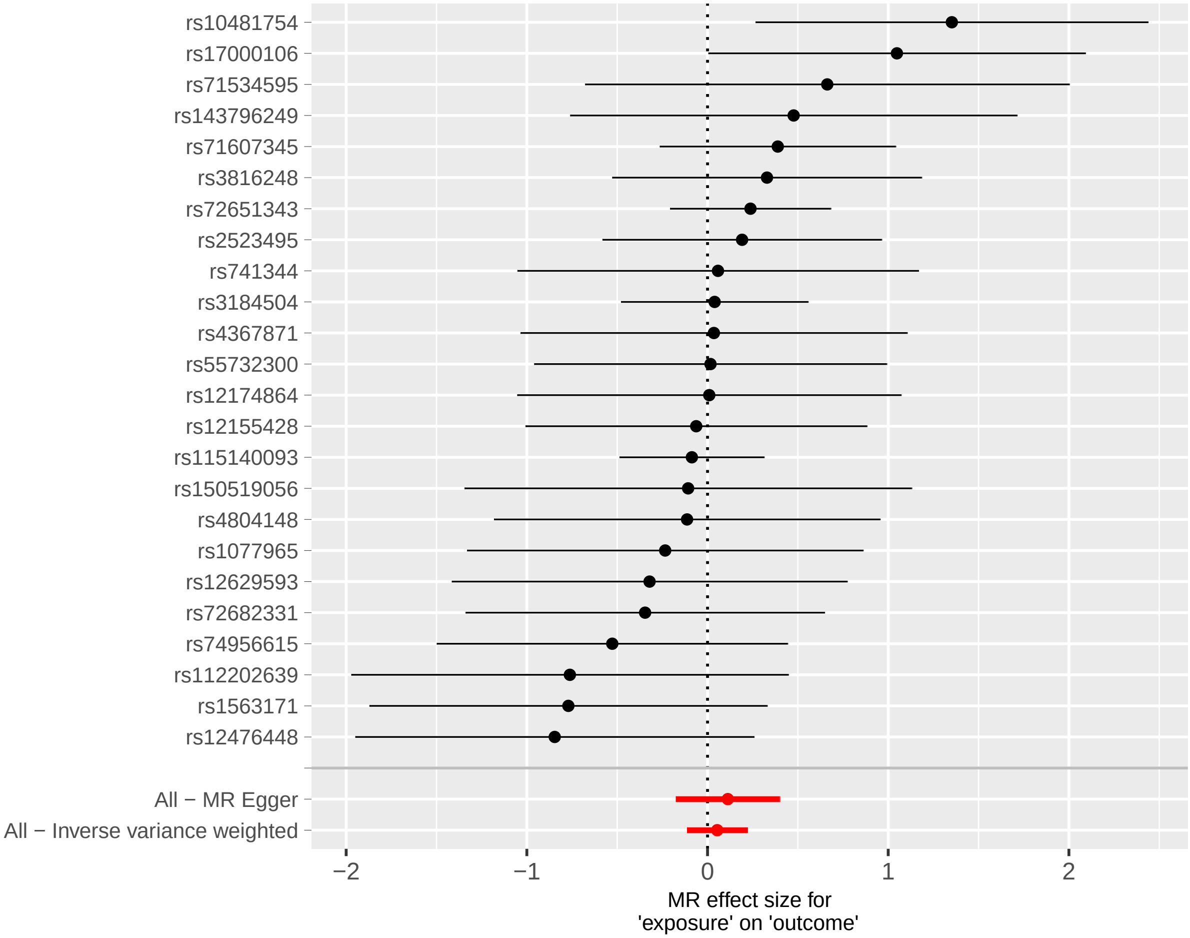

rs10481754
rs17000106
rs71534595
rs143796249
rs71607345
rs3816248
rs72651343
rs2523495
rs741344
rs3184504
rs4367871
rs55732300
rs12174864
rs12155428
rs115140093
rs150519056
rs4804148
rs1077965
rs12629593
rs72682331
rs74956615
rs112202639
rs1563171
rs12476448
All − MR Egger
All − Inverse variance weighted
−2
−1
0
1
2
MR effect size for
'exposure' on 'outcome'
